# Necrostatin-1 Ameliorates Peripheral Nerve Injury-Induced Neuropathic Pain by Inhibiting the RIP1/RIP3 Pathway

**DOI:** 10.3389/fncel.2019.00211

**Published:** 2019-05-15

**Authors:** Ying-Xia Liang, Nan-Nan Wang, Zhi-Yu Zhang, Zhao-Dong Juan, Can Zhang

**Affiliations:** ^1^Medicine and Health Key Laboratory of Clinical Anesthesia, Department of Anesthesiology, Weifang Medical University, Weifang, China; ^2^Genetics and Aging Research Unit, Department of Neurology, MassGeneral Institute for Neurodegenerative Disease, Massachusetts General Hospital, Harvard Medical School, Charlestown, MA, United States; ^3^Department of Microsurgery, Shouguang People’s Hospital, Weifang, China

**Keywords:** necroptosis, necrostatin-1, neuropathic pain, inflammatory cytokines, hyperalgesia, peripheral nerve injury

## Abstract

Necrostatin-1 is an inhibitor of necroptosis, a form of programmed cell death that has been reported to be involved in various neurological diseases. Presently, the role of necroptosis in neuropathic pain induced by peripheral nerve injury is still unclear. This study was focused on investigating the potential effects of necroptosis in the development and progression of neuropathic pain in a rat model and the possible neuroprotective effects of necrostatin-1 in neuropathic pain. The results indicated that the necroptosis-related proteins RIP1 and RIP3 significantly increased postoperation in the spinal cord in a neuropathic pain model and peaked 7 days postoperation, which was consistent with the time-dependent changes of hyperalgesia. Additionally, we found that peripheral nerve injury-related behavioral and biochemical changes were significantly reduced by necrostatin-1. In particular, hyperalgesia was attenuated, and the levels of RIP1 and RIP3 were decreased. Furthermore, the ultrastructure of necrotic cell death and neuroinflammation were alleviated by necrostatin-1. Collectively, these results suggest that necroptosis is an important mechanism of cell death in neuropathic pain induced by peripheral nerve injury and that necrostatin-1 may be a promising neuroprotective treatment for neuropathic pain.

## Introduction

Neuropathic pain is a severe disease caused by lesions in the nervous system and may persist for months to years, even after the primary disease or injury has healed (Haanpaa et al., 2011). It is a devastating disorder and it reduces the quality of life of millions of people around the world (Gaskin and Richard, 2012). Current analgesic drugs, such as non-steroidal anti-inflammatory drugs, tricyclic antidepressants, and opioids, lack satisfactory efficacy in controlling disease progression, and most of them alleviate symptoms by inhibiting neuronal activity. In addition, these drugs are associated with serious toxicity and dose-limiting side effects, such as dependence, addiction, dizziness, constipation, and blurred vision (Ji et al., 2014; Fernandes et al., 2018). Therefore, it is crucial to elucidate the mechanisms of neuropathic pain and to explore better therapeutic interventions.

The pathogenesis of neuropathic pain is complex and includes peripheral and central neuronal sensitization as well as neuroinflammation mediated by inflammatory mediators and chemokines (Ristoiu, 2013). Accumulating evidence has demonstrated that neuroinflammation plays a vital role in the induction and maintenance of chronic pain (Woolf, 2011; Finnerup et al., 2016; Colloca et al., 2017). In particular, neuroinflammation in neuropathic pain involves the infiltration of immune cells, activation of glial cells and production of inflammatory mediators in the peripheral and central nervous systems (e.g., dorsal root ganglia and spinal cord dorsal horn) (Hansson, 2015; Wallach et al., 2016; Chen and Lu, 2017). Recent progress has indicated that neuroinflammation is responsible for generating and sustaining the sensitization of nociceptive neurons that lead to chronic pain (Kawasaki et al., 2008; Rivest, 2009). Therefore, targeting the processes and molecules that are involved in neuroinflammation could lead to better treatments for chronic pain (Ji et al., 2014).

Recently, studies have reported that necrotic cell death leads to the release of cellular components that may facilitate inflammation in a programmed manner through distinct sets of signaling mechanisms. These studies have shown that necroptosis, a form of programmed cell death, may be a trigger of inflammation involved in pain (Pasparakis and Vandenabeele, 2015; Newton and Manning, 2016; Wallach et al., 2016; Koehler et al., 2017; Shan et al., 2018). Specifically, necroptosis is a type of caspase-independent necrotic cell death that can be mediated by the kinases RIP1/RIP3 (Shan et al., 2018). Importantly, the kinase activity of RIP1 is critical to mediate necroptosis (Holler et al., 2000; Degterev et al., 2008), and RIP3, a RIP1 family member, is also crucial for necroptosis (Christofferson and Yuan, 2010). RIP1 and RIP3 may interact through their RIP isoform interaction motifs (RHIM) (Sun et al., 2002), and this interaction may lead to a specific necroptotic signaling pathway as a part of the conventional necroptosis pathway (Cho et al., 2009; He et al., 2009).

In addition to pain, necroptosis may participate in a variety of other pathologic conditions, including neurodegenerative disease, sepsis, and ischemic reperfusion injury (Cho, 2018). Due to these critical roles, effort has been focused on identifying its regulatory molecules (Feoktistova and Leverkus, 2015). Recently, 5-(1H-indol-3-ylmethyl)-2-thiohydantoin 1, named necrostatin-1 (Nec-1), which specifically inhibits RIP1 and RIP-mediated necroptosis, was identified (Degterev et al., 2005). Particularly, necrostatin-1 is a small-molecule that readily crosses the blood-brain barrier (Jagtap et al., 2007; Zhu et al., 2011). Studies on necroptosis have shown that necrostatin-1 is an effective necroptosis inhibitor that can protect cellular structures under necroptosis-related pathological conditions. For example, necrostatin-1 may inhibit necroptosis and exert protective effects after spinal cord injury and may protect against Alzheimer’s disease (Caccamo et al., 2017). It can reduce pro-inflammatory cytokines, oxidative stress and tissue damage and can restore neurological function by mitigating mitochondrial damage and maintaining energy homeostasis (Wang et al., 2017; Yang et al., 2017).

At present, little is known about the effects of necrostatin-1 on neuropathic pain and its management. In this study, we aimed to explore the role of necroptosis in neuropathic pain induced by peripheral nerve injury in rats and to investigate the potential neuroprotective effects of necrostatin-1. The results from this study will be useful in demonstrating whether necrostatin-1 may provide functional improvement in neuropathic pain and whether inhibiting necroptosis can be a target for neuropathic pain management.

## Materials and Methods

### Animal

All animal experiments were approved by the Institutional Animal Care and Use Committee and performed following the NIH Guide for the Care and Use of Laboratory Animals. The study protocols were designed to minimize animal numbers and suffering. Adult male Sprague-Dawley rats (weight: 230 ± 10 g, age: 8-week-old) were purchased from Sibeifu Biotechnology Co., Ltd. (China). Rats were housed in a standard room with controlled temperature (20–22°C) and humidity (55–60%) under a12-h light/dark cycle with free access to water and food.

### Chronic Constriction Injury (CCI) Model

All rats were anesthetized with 40 mg/kg sodium pentobarbital intraperitoneally. In brief, the right sciatic nerve was separated at the midthigh level and loosely ligated at 1 mm intervals using four chromic catgut sutures. It was ensured that the hind limb briefly twitched and that the nerve was not squeezed too tightly as to prevent blood flow around the nerve. The muscles and skin were sutured in layers. In Sham-operated rats, the right sciatic nerve was exposed but not ligated (Bennett and Xie, 1988). The CCI rats were tested, and those that exhibited significant mechanical and thermal hypersensitivity reactions were used for further experiments. All surgical procedures were performed under aseptic conditions.

### Experimental Design

For the behavioral experiments, the surgical rats were randomly divided into the following groups (*n* = 10): Sham, CCI, Sham+DMSO, CCI+DMSO, CCI+400 μg/ml Necrostatin-1, CCI+200 μg/ml Necrostatin-1, and CCI+100 μg/ml Necrostatin-1. Rats in the CCI+necrostatin-1 group were treated intraperitoneally with necrostatin-1. Necrostatin-1 (25 mg, Sigma, United States) was dissolved in dimethyl sulfoxide (DMSO). Rats in the CCI+DMSO group were treated with an equal volume of DMSO in the same manner. The baseline thresholds were tested 1 day before surgery. The changes in mechanical withdrawal threshold (MWT) and thermal withdrawal latency (TWL) were examined at 1, 3, 5, 7, 10, 14, and 21 days after surgery. In the Western blot experiment, the expression of necroptosis-related proteins was measured. The subsequent experiments included transmission electron microscopy, propidium iodide (PI) labeling and an enzyme-linked immunosorbent assay and were used to evaluate the protective effect of necrostatin-1 against hyperalgesia. The animals were divided into the following three groups (*n* = 6): Sham group, CCI group, and CCI+necrostatin-1. All rats were sacrificed 7 days after surgery.

### Behavioral Tests

#### Assessment of Mechanical Allodynia

Mechanical withdrawal threshold was tested with the up-down method described previously (Dixon, 1980; Chaplan et al., 1994; Zhang et al., 2018). On each test day, rats were placed in a transparent plastic box on a wire mesh floor at least 30 min prior to the test period. The baseline threshold of the hind paw of all rats was tested 1 day before surgery. The mechanical behavioral test used a set of von Frey hairs (Ugo Basile, Italy) with a logarithmic increase in stiffness ranging from 3.61 (0.41 g) to 5.18 (15.14 g). The 2 g stimulus was first applied to the middle of the hind paw. A quick withdrawal or claw retraction in response to the stimulus was considered a positive response. If there was no paw withdrawal, the next stronger stimulus was chosen. Otherwise, a weaker stimulus was applied. A series of tests were conducted 1, 3, 5, 7, 10, 14, and 21 days after surgery.

#### Assessment of Thermal Hyperalgesia

Thermal withdrawal latency was tested according to the method described by Hargreaves et al. (1988). On each test day, rats were placed into individual plastic cages on a glass floor for at least 30 min prior to the test period. The baseline threshold of the hind paw of all rats was tested 1 day before surgery. The thermal behavioral test was performed using a plantar test device (PL-200 radiant heat apparatus, Timing Technology and Market Corporation, China). In brief, a radiant heat source was targeted at the plantar surface of the hind paw. The hind paw was tested alternately with greater than 5-min intervals between consecutive tests. A maximal cutoff of 25 s was used to prevent tissue damage (Na et al., 2008). The three measurements of latency were averaged as the result of each test.

### Propidium Iodide Labeling

Propidium iodide labeling was used to investigate cytosolic membrane permeability and was modified slightly from previous methods (Chen et al., 2017). In brief, the rats were injected intraperitoneally with PI (1 mg/kg, Sigma, United States) diluted in physiological saline. After 1 h, rats were perfused with physiological saline followed by 4% paraformaldehyde under anesthesia. The L4–L6 segments of the spinal cord were isolated, and the lumbar enlargement segments of the spinal cord were sectioned transversely and postfixed overnight with 4% paraformaldehyde, followed by dehydration with 30% sucrose. Spinal cord sections (10 μm thick) were transversely cut with a cryostat (Leica CM1850). The sections were washed with PBS, covered with 4′,6-diamidino-2-phenylindole, and then immediately photographed using a fluorescence microscope (BX50, Olympus Co., Japan). The quantification of PI-positive cells was evaluated by another researcher who was blinded to the experimental design. Six fields per sample were examined at random.

### Transmission Electron Microscopy

The spinal cord tissue was prepared for transmission electron microscopy as described previously (Wang et al., 2017). Rats were perfused with PBS containing 4% paraformaldehyde, and the lumbar enlargement segments of the spinal cord were separated and fixed with 2.5% glutaraldehyde. Tissue fragments were transferred to 1% osmic acid and then dehydrated in a gradient alcohol solution and 100% acetone for postfixation. Processed tissues were embedded with Epon 812 and sectioned at 50 nm. Finally, sections were stained with 3% uranium citrate and lead acetate and imaged by a HITACHI transmission electron microscope (HT7700, Japan).

### Western Blotting

Western blot analysis was performed using a previously described method (Liang et al., 2012). Proteins in the lumbar enlargement segments of the spinal cord were extracted using a tissue lysis kit (Solarbio, China). The tissue homogenate was centrifuged at 12,000 rpm for 30 min at 4°C. The protein concentration was determined with a BCA protein assay kit (Cwbio, China). Protein (40 μg) was loaded and separated by 12% SDS-PAGE gel and then transferred to a PVDF membrane (Millipore, Billerica, MA, United States). The membrane was incubated with RIPK1 (1:1000, Cell Signaling, United States, Catalog #3493), RIPK3 (1:1000, Abcam, United States, Catalog #62344), NF-κB p56 (1:1000, Bioss Antibodies, China, Catalog # bsm-33117M), phospho-NFκB p56 (1:1000, Bioss Antibodies, China, Catalog # bs-0982R), and β-actin (1:20000, Proteintech, China, Catalog #66009-1) antibodies overnight at 4°C. Next, the membranes were incubated with horseradish peroxidase-conjugated secondary antibodies (1:5000, Proteintech, China) for 2 h at room temperature. The bands were visualized using ECL Western blotting detection reagents (Thermo Fisher Scientific, United States) and tested with a chemiluminescence system (Tanon-5200, Tanon, China). The band intensities were quantified using Image-Pro Plus analysis software.

### Enzyme-Linked Immunosorbent Assay

Blood samples were collected from the heart and were centrifuged for 15 min at 2500 rpm at 4°C. The supernatants were collected separately. The protein in the lumbar enlargement segments of the spinal cord was extracted using a tissue lysis kit (Solarbio, China). The lumbar enlargement segments were homogenized and centrifuged for 30 min at 12000 rpm at 4°C. The supernatant was collected. The levels of IL-1β (Multi Sciences, China), TNF-α and SP (Westang, China) in the serum and in the spinal cord tissues were determined using ELISA kits according to the manufacturer’s protocol. Optical density (OD) values were measured at 450 and 630 nm using a microplate reader (Bio-Rad, Japan). We calculated the concentration of cytokines in different groups by comparing the values of the samples with the standard curve.

### Statistical Analysis

Data were presented as the mean ± SEM. For the behavioral tests, two-way ANOVA with repeated measures was used for comparison between groups. The results of Western blot and ELISA were analyzed by one-way ANOVA with the least significant difference test (LSD-t). The statistical analysis was performed using SPSS 19.0. *p* < 0.05 was considered statistically significant.

## Results

### Necrostatin-1 Relieved Neuropathic Hyperalgesia Induced by CCI

**FIGURE 1:**
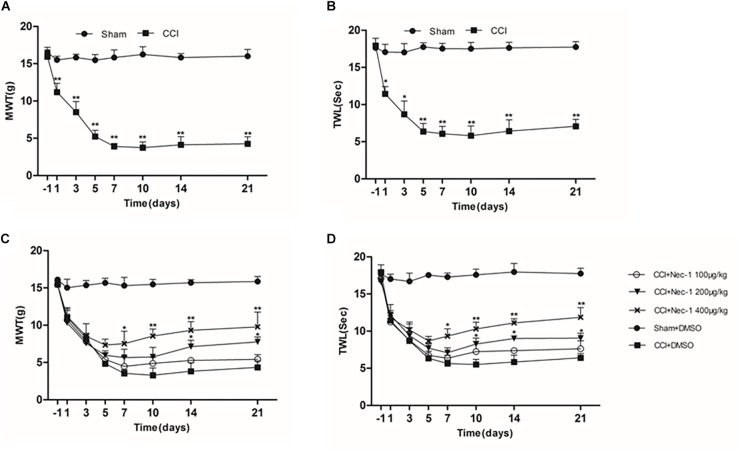
Effects of necrostatin-1 on allodynia in a rat model of neuropathic pain. The development and maintenance of mechanical **(A)** and thermal **(B)** allodynia was demonstrated in rats with peripheral nerve injury, and necrostatin-1 attenuated both mechanical allodynia **(C)** and thermal hypersensitivity **(D)**. Mechanical withdrawal threshold (MWT) was measured by the von Frey test, and the paw withdrawal thermal latency (TWL) was measured by thermal testing, each at different time points. *N* = 10 for each group; ^∗^*p* < 0.05, ^∗∗^*p* < 0.01 compared to the CCI+DMSO group on the same postoperative day. MWT, mechanical withdrawal threshold; TWL, thermal withdrawal latency.

The effect of necrostatin-1 on neuropathic pain was examined using a rat sciatic nerve chronic constriction injury (CCI) model. Briefly, the adult male Sprague-Dawley rats were randomly divided into the following two groups (*n* = 10): the Sham group (nerve exposed without ligation) and the CCI-operated group (CCI). As shown in Figure 1A,B, the CCI group rats developed mechanical and thermal allodynia after surgery (^∗^*p* < 0.05), unlike the Sham group rats. As expected, the mechanical and thermal allodynia started 1 day after surgery and persisted for 3 weeks after surgery.

Then, we investigated whether necrostatin-1 attenuated the behavioral defects in the CCI group. The CCI group rats received different doses of necrostatin-1 or DMSO (vehicle) by intraperitoneal (IP) administration. Specifically, the animals were injected intraperitoneally with different doses of necrostatin-1 (100, 200, or 400 μg/kg) once a day for 21 days. The behavioral tests were performed at 1, 3, 5, 7, 10, 14, and 21 days after surgery. We showed that rats treated with necrostatin-1 were dose-dependently relieved of mechanical and thermal allodynia from 7 to 21 days postoperation (^∗^*p* < 0.05), unlike rats in the CCI group. Specifically, as shown in Figure 1C, CCI rats displayed hyperalgesia and a low mechanical withdrawal threshold (MWT). In contrast, rats treated with necrostatin-1 (200 and 400 μg/ml) showed reduced mechanical allodynia and displayed increased MWT at 7, 10, 14, and 21 days after surgery (^∗^*p* < 0.05). Additionally, compared to rats in the CCI group, rats treated with necrostatin-1 exhibited significantly prolonged thermal withdrawal latency (TWL) (^∗^*p* < 0.05), as shown in Figure 1D. In particular, the CCI rats displayed hyperalgesia and a shorter TWL. In contrast, necrostatin-1 dose-dependently decreased thermal allodynia at 7, 10, 14, and 21 days after surgery (^∗^*p* < 0.05). The 400 μg/kg dose of necrostatin-1 demonstrated the highest level of behavioral improvement and was therefore selected for subsequent experiments.

### Increased Expression of RIP1 and RIP3 in the Spinal Cord of a Neuropathic Pain Model

**FIGURE 2:**
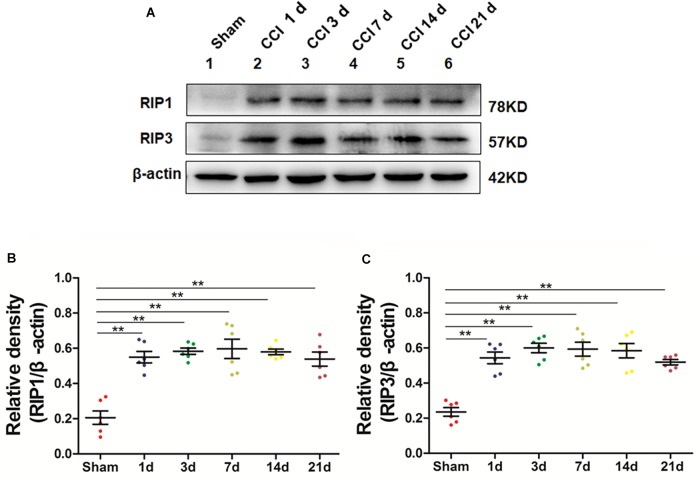
Upregulation of the necroptosis-related proteins RIP1/RIP3 in the spinal cord in rats with neuropathic pain. **(A)** Time course changes of RIP1 and RIP3 proteins in the spinal cord induced by peripheral nerve injury as detected by Western blot. **(B,C)** Quantitative analysis of Western blotting for the proteins RIP1 **(B)** and RIP3 **(C)**. The *y*-axis indicates the relative density of these proteins (*n* = 6 per group). ^∗^*p* < 0.05, ^∗∗^*p* < 0.01 compared to the Sham group.

We next explored whether necroptosis was induced in our rat model of CCI. Specifically, we performed Western blot analysis to detect the levels of the necroptosis-related proteins RIP1 and RIP3 in the spinal cord at different time points postoperation. The expression of RIP1 was significantly increased (^∗^*p* < 0.05; vs. Sham) beginning the first day after surgery, a change that lasted for 21 days postoperation in our investigation, as shown in Figure 2A–C. Additionally, the levels of RIP3 increased simultaneously with RIP1 compared to those of the Sham group (^∗^*p* < 0.05). Thus, these results showed that necroptosis was induced in our rat model of CCI.

### Necrostatin-1 Inhibited RIP1/RIP3-Mediated Necroptosis in Rats With Neuropathic Pain

**FIGURE 3:**
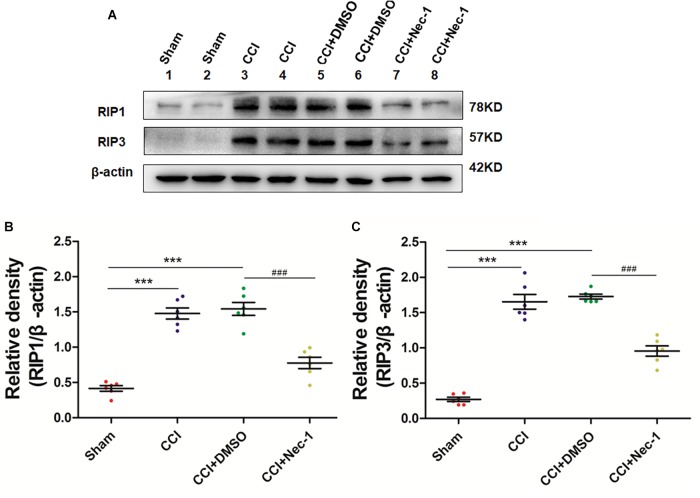
Necrostatin-1 reduced the levels of necroptosis-related RIP1/RIP3 proteins after CCI injury. The levels of RIP1/RIP3 proteins in rats from various groups, including Sham (nerve exposed without ligation); CCI; CCI+DMSO and CCI+Necrostatin-1 groups, were investigated through Western blot analysis **(A)**. Quantitative results of the Western blot analyses (**B**, RIP1; **C**, RIP3). The spinal cord was removed 90-min after necrostatin-1 administration. The *y*-axis indicated the relative density of these proteins (*n* = 6 per group). ^∗∗∗^*p* < 0.001 versus the sham group; ^###^*p* < 0.001 versus the CCI+DMSO group.

Next, we aimed to investigate the potential effect of necrostatin-1 on necroptosis in the rats of our neuropathic pain model. We utilized Western blot analysis of the protein levels of RIP1 and RIP3 in the samples of animals from different groups, including Sham (nerve exposed without ligation), CCI (CCI-operated), CCI with DMSO (CCI+DMSO) and CCI with 400 μg/kg necrostatin-1 (CCI+Nec-1) groups. We showed that the levels of RIP1 and RIP3 in the spinal cord of the CCI group were upregulated compared to those of the Sham group, as expected. We also showed that the RIP1 and RIP3 levels in the CCI+Nec-1 group were decreased compared to those in the CCI+DMSO group (^∗∗^*p* < 0.01), as shown in Figure 3. Thus, the results suggested that necrostatin-1 significantly inhibited RIP/RIP3-mediated necroptosis in the spinal cord of rats with neuropathic pain.

### Necrostatin-1 Decreased the Number of PI-Positive Cells in Rats With Neuropathic Pain

**FIGURE 4:**
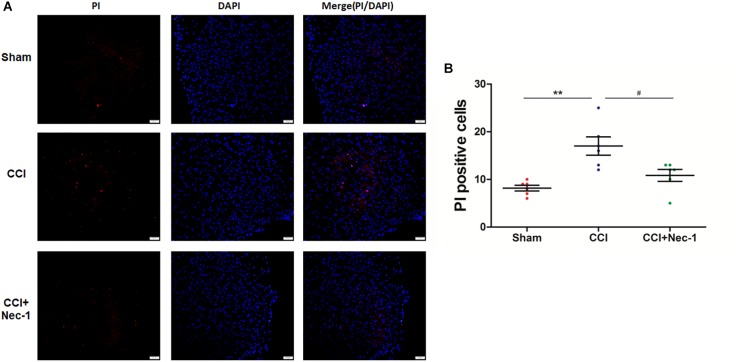
Necrostatin-1 decreased the number of necrotic cell death PI-positive cells induced by CCI. Immunohistochemistry was utilized to detect PI-stained cells that underwent necrotic cell death in rats from different groups, including Sham (nerve exposed without ligation), CCI and CCI+Nec-1 groups. **(A)** Magnification 200×, scale bar = 50 μm. **(B)** Quantification of PI-positive cells. Data are represented as the mean ± SEM. ^∗∗^*p* < 0.01 versus the Sham group; ^#^*p* < 0.05 versus the CCI group. PI, propidium iodide.

Next, we performed immunofluorescence analysis to further characterize the cellular viability changes in rats with neuropathic pain described above (Cho et al., 2009). In particular, we performed a previously reported PI-staining method to observe the effect of necrostatin-1 in rats with neuropathic pain, focusing on 7 days postoperation. We found that very few PI-positive cells were identified from the spinal cord of rats in the Sham group, as expected. In contrast, PI-positive cells in the spinal cord of rats with neuropathic pain significantly increased and were reduced after treatment with 400 μg/kg necrostatin-1. As shown in Figure 4, the number of PI-positive cells in the CCI group was 17 ± 2.1, which was significantly higher than that of the Sham group (8.2 ± 1.1; ^∗∗^*p* < 0.01). However, rats treated with necrostatin-1 exhibited a significantly decreased number of PI-positive cells (10.8 ± 1.6; #*p* < 0.05) compared to those in the CCI group.

### Necrostatin-1 Relieved Necrotic Cell Death After Surgery

**FIGURE 5:**
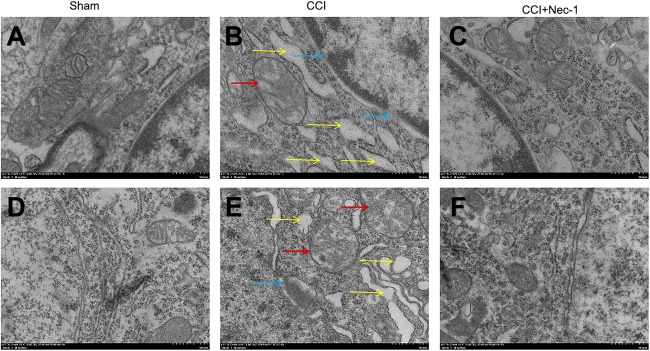
Representative microphotographs of transmission electron microscopy in the spinal cord of rats with peripheral nerve injury. **(A–C)** Spinal cord neurons and **(D–F)** glial cells are shown. **(A,D)** Spinal cord neurons and glial cells showed normal cell morphology, intact nuclear membranes and normal endoplasmic reticulum structure in the Sham group. **(B,E)** The CCI group animals displayed typical features of necrosis, including membrane integrity breakdown, nuclear membrane dissolution (denoted by blue arrows), vacuolation (denoted by red arrows), and endoplasmic reticulum dilation (denoted by yellow arrows). **(C,F)** The necrotic changes in the CCI group were ameliorated compared to the CCI+necrostatin-1 group. The spinal cord neurons and glial cells presented improved nuclear membrane integrity, less mitochondrial swelling, and vacuolar-like changes, and reduced endoplasmic reticulum expansion. Magnification (5,000×), scale bar=2 μm.

Furthermore, we investigated the effects of necrostatin-1 on rats with neuropathic pain using transmission electron microscopy. Necrosis was characterized by the presence of typical features of necrotic cells under transmission electron microscope examination (Chen et al., 2017). In the Sham group (Figure 5A,D), spinal cord neurons and glial cells showed almost normal cell morphology, intact nuclear membranes, and normal endoplasmic reticulum structure. In contrast, neurons, and glial cells in the spinal cord of rats with neuropathic pain exhibited typical necrotic changes, including nuclear membrane dissolution, vacuolation, the breakdown of membrane integrity, and significantly dilated endoplasmic reticula (Figure 5B,E). However, these conditions were ameliorated in the CCI+necrostatin-1 group. After treatment with necrostatin-1, spinal cord neurons and glial cells exhibited nuclear membranes with improved integrity comparable to those of rats in the Sham group, as well as reduced mitochondrial swelling, vacuolar-like changes and endoplasmic reticulum expansion (Figure 5C,F). These results, together with the findings from PI-staining, supported the potential for necrostatin-1 to attenuate necrotic cell death in the spinal cord of rats with neuropathic pain.

### Necrostatin-1 Decreased the Release of Inflammatory Cytokines and Substance P by NF-κB in Rats With Neuropathic Pain

**FIGURE 6:**
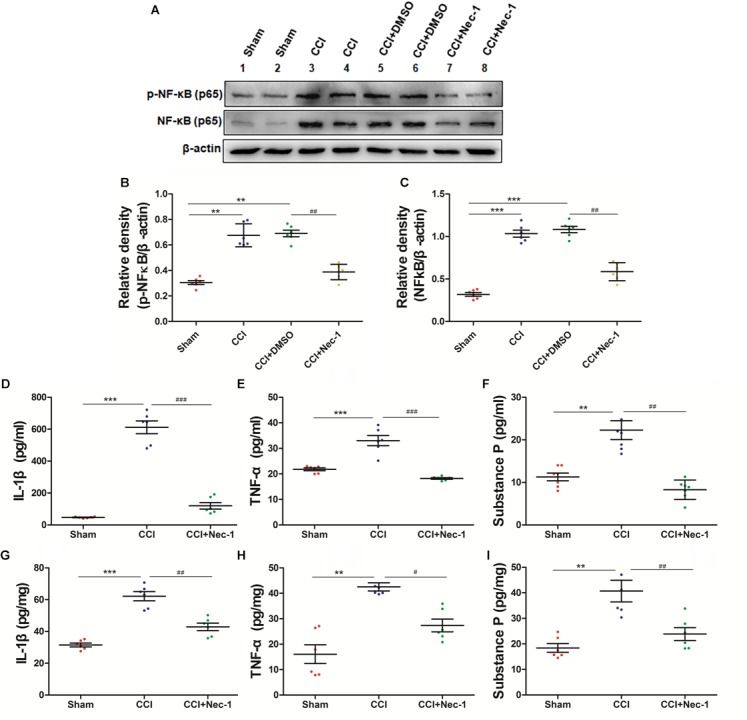
Necrostatin-1 attenuated neuroinflammatory changes in a rat neuropathic pain model. Biochemical studies, including Western blot analysis **(A–C)** and ELISA **(D–I)**, were performed to determine the neuroinflammatory proteins involved in neuropathic pain. **(A)** Changes in NF-κB p65 after necrostatin-1 treatment. **(B,C)** Quantitative results of the Western blot analyses. The levels of proinflammatory cytokines in the serum **(D–F)** and spinal cord tissue **(G–I)** were investigated by ELISA. The concentrations of IL-1β **(D,G)**, TNF-α **(E,H)**, and substance P **(F,I)** were significantly decreased after treatment with necrostatin-1 (*n* = 6 for each group). ^∗∗^*p* < 0.01, ^∗∗∗^*p* < 0.001 versus the Sham group; ^#^*p* < 0.05, ^##^*p* < 0.01, ^###^*p* < 0.001 versus the CCI group.

To further explore whether necrostatin-1 affects neuroinflammation in neuropathic pain, we focused on the role of necrostatin-1 in proinflammatory NF-κB signaling pathways in neuropathic pain. The NF-κB pathway is an important signaling pathway for the release of inflammatory mediators in cells (Lin et al., 2004; Todd, 2010). The results showed that NF-κB was upregulated in the spinal cord of the CCI group, whereas this effect was significantly decreased after necrostatin-1 treatment, as shown in the CCI+necrostatin-1 group (^∗^*p* < 0.05). Our results also showed that the inflammatory cytokines TNF-α and IL-1β and the neurotransmitter substance P were upregulated in the serum and spinal cord tissue of the CCI group. As shown in Figure 6, the levels of TNF-α, IL-1β and the neurotransmitter substance P in the CCI group were significantly higher than those in the Sham operation group (^∗∗^*p* < 0.01). However, compared to the rats in the CCI group, rats treated with necrostatin-1 exhibited significantly reduced levels of inflammatory cytokines and substance P (^∗^*p* < 0.05), indicating a significant decrease in neuroinflammation in the spinal cord of rats with neuropathic pain after administration of necrostatin-1.

## Discussion

Millions of people around the world suffer from neuropathic pain, which places a heavy burden on families and society. However, there is currently no satisfactory treatment (Haanpaa et al., 2011). Necroptosis, a form of programmed cell death, is associated with neuropathological diseases, such as Alzheimer’s disease (AD), multiple sclerosis (MS), and Parkinson’s disease (PD) (Ofengeim et al., 2015; Wu et al., 2015; Caccamo et al., 2017); however, there is no report on the role of necroptosis in neuropathic pain. In this study, we explored whether necroptosis is involved in the development and maintenance of neuropathic pain. We first investigated RIP1/RIP3-mediated necroptosis in the spinal cord by measuring mechanical and thermal hyperalgesia as well as the necrotic cell death and inflammatory response in neuropathic pain. In addition, treatment with necrostatin-1 reduced nerve damage, relieved hyperalgesia, and reduced the inflammatory response. Our results suggest that necroptosis may be an important pathological mechanism of hyperalgesia induced by peripheral nerve injury and that necrostatin-1 has a protective effect on neuropathic pain by inhibiting necroptosis.

Chronic pain is typically characterized by hyperalgesia, which is an increased response to noxious thermal and mechanical stimuli, coupled with allodynia. The spinal dorsal horn plays an important role in central sensitization in neuropathic pain. Sensory nerves transmit noxious thermal and mechanical stimuli information from the body to the spinal dorsal horn, and the spinal dorsal horn integrates and expands the noxious stimuli and then transmits this information to the brain (Todd, 2010; Ji et al., 2014, 2016; West et al., 2015). In this study, the results indicated that the key necroptosis-related proteins RIP1 and RIP3 increased in the spinal cord of rats with neuropathic pain compared to rats in the sham group. As shown, the rats with neuropathic pain developed intense mechanical and thermal allodynia that continued for 21 days after surgery. The neuropathic pain group showed a significant increase in RIP1 and RIP3 1, 3, 7, 14, and 21 days after surgery. In addition, the animals with high levels of the necroptosis-related proteins RIP1 and RIP3 exhibited significant allodynia in behavioral tests. It has been reported that pro-death signaling molecules, such as factor-related apoptosis ligands and TNF-α, bind to their death receptors to promote the assembly of necrosomes and trigger necroptosis (Degterev et al., 2005). Consistently, peripheral nerve injury caused an increase in the production of the inflammatory cytokine TNF-α in our study. Combined with these results, it indicates that necroptosis is activated in and is closely related to neuropathic pain induced by peripheral nerve injury.

Necrostatin-1, a potent inhibitor of RIP1, is able to inhibit the activation of RIP1 and subsequently block the formation of necrosomes, ultimately blocking necroptosis. We further explored the protective effects of necrostatin-1 on neuropathic pain. The results showed that the necroptosis-related proteins RIP1 and RIP3 were reduced after necrostatin-1 treatment. In addition, our results confirmed that necrostatin-1 reduces hyperalgesia in mechanical and thermal tests and promotes the recovery of neurobehavioral function. Neuronal cell death and inflammatory responses were also significantly improved after necrostatin-1 treatment. Consistent with our results, necrostatin-1 has been shown to inhibit necrotic neuronal death in rat models of cerebral infarction and traumatic brain injury, associated with improved pathological defects (Kusaka et al., 2004; Lin et al., 2004; You et al., 2008).

Our study identified a new molecular mechanism, i.e., necroptosis, underlying neuropathic pain in animals and further characterized its impacts in relieving neuropathic defects by a specific pharmacological inhibitor of necroptosis. We further investigated cell viability using PI-staining analysis and confirmed necrotic cell death through transmission of electron microscopy in these animals. Necroptosis represents a mode of programmed cell death which can be mediated by RIP1/RIP3 and other proteins, in contrast to the necrotic cell death and conventional apoptotic cell death. Necroptosis is usually accompanied by plasma membrane rupture, organelle swelling, and inflammatory cell infiltration, which usually promotes inflammatory responses (Pasparakis and Vandenabeele, 2015; Dhuriya and Sharma, 2018). Specifically, it may act as an initiation signal that contributes to the amplification and chronicity of inflammation, and leads to the pathogenesis of chronic inflammatory disease (Pasparakis and Vandenabeele, 2015).

Notably, the inflammatory response has been recognized as an important mechanism of the pathogenesis of neuropathic pain (Wei et al., 2008; Ji et al., 2014, 2016). NF-κB, the pro-inflammatory transcription factor, plays a key role in the process of the inflammatory response and is a central component of pro-inflammatory signaling (Lin et al., 2004; Todd, 2010). Substance P, a major neuropeptide, induces pain by stimulating proximal effectors to sensitive nociceptors (Austin and Moalem-Taylor, 2010). The cytokines TNF-α and IL-1β were increased in patients suffering neuropathic pain in the serum, and were associated with the extent of pain and the severity of disease progression (Leung and Cahill, 2010).

Our current investigation identifies and characterizes the therapeutic effects and molecular mechanisms by which necrostatin-1 ameliorates neuropathic, focusing on the necroptotic processes and inflammatory responses. Particularly, we investigated the expression of NF-κB signal and its downstream effector molecules, IL-1β, TNF-α, and substance P. Our results showed that activation of necrotic apoptosis in the spinal cord was accompanied by increased expression of NF-κB and the release of pro-inflammatory cytokines and substance P in our CCI animal model; while neuroinflammation was relieved and substance P was reduced in the serum and spinal cord tissue by necrostatin-1. Notably, our results were in line with other findings suggesting that circulating inflammatory proteins in the peripheral system may be important biomarkers and provide important mechanisms in the pathogenesis of neuropathic pain (Safieh-Garabedian et al., 2018). Thus, our data suggested that necrostatin-1 may display anti-nociceptive effects through combined necroptotic- and immune-regulatory mechanisms on both the central nervous system and the peripheral system.

There are certain limitations to our current research. In our study, the cell types that underwent necroptosis have not been identified. It has been reported that the RIP1/RIP3 were expressed in the dorsal root ganglion (DRG), spinal cord and brain (Fan et al., 2016; Pu et al., 2018). Our findings warrant future studies to characterize the expression of RIP1 and RIP3 in different cell types in these areas and related molecular mechanisms underlying neuropathic pain. PI-staining may indicate cell viability but may not differentiate cell death of various causes and can appear positive in necrotic cell death, programmed cell death, and other mechanisms. Furthermore, we have not focused on the impacts of DRG in our neuropathic pain animals which contains the sensory neurons that primarily transmit sensory information from the periphery to the central nervous system (Krames, 2014). We anticipate that DRG in our animals may undergo neuropathic pain-related changes, including necroptosis and inflammatory alterations, which should be investigated in future studies. Moreover, necroptosis can be mediated by other proteins, e.g., the mixed lineage kinase domain-like pseudokinase (MLKL). It will be interesting to assess this protein in our neuropathic pain animals. Collectively, our data highlights the critical roles of necroptosis and warrant future studies in these specific areas for their impacts on neuropathic pain.

## Conclusion

We first investigated the effects of necroptosis in the spinal cord of neuropathic pain and found that blocking necroptosis effectively relieved hyperalgesia, promoted behavioral recovery and decreased tissue damage. These results suggest that necroptosis may be an important pathogenic mechanism of neuropathic pain. Furthermore, our data supports that necrostatin-1 might be a novel therapeutic strategy to alleviate neuropathic pain induced by peripheral nerve injury.

## Data Availability

All datasets generated for this study are included in the manuscript and/or the supplementary files.

## Ethics Statement

All animal experiments were carried out in accordance with the recommendations of the NIH Guide for the Care and Use of Laboratory Animals. The protocol was approved by the Animal Care and Use Committee of Weifang Medical University.

## Author Contributions

Y-XL and Z-DJ contributed to the conception and design of the study. N-NW and Z-YZ performed the experiments. N-NW performed the statistical analysis. Y-XL wrote the first draft of the manuscript. CZ interpreted the results and reviewed and revised the manuscript. All authors contributed to manuscript revision and read and approved the submitted version.

## Conflict of Interest Statement

The authors declare that the research was conducted in the absence of any commercial or financial relationships that could be construed as a potential conflict of interest.
